# Resolving the spin reorientation and crystal-field transitions in *Tm*FeO_3_ with terahertz transient

**DOI:** 10.1038/srep23648

**Published:** 2016-03-24

**Authors:** Kailin Zhang, Kai Xu, Xiumei Liu, Zeyu Zhang, Zuanming Jin, Xian Lin, Bo Li, Shixun Cao, Guohong Ma

**Affiliations:** 1 Department of Physics, Shanghai University, Shanghai 200444, China; 2 Key Laboratory of Polar Materials and Devices, East China Normal University, Shanghai 200241, China

## Abstract

Rare earth orthoferrites (RFeO_3_) exhibit abundant physical properties such as, weak macroscopic magnetization, spin reorientation transition, and magneto-optical effect, especially the terahertz magnetic response, have received lots of attention in recent years. In this work, quasi-ferromagnetic (FM) and quasi-antiferromagnetic (AFM) modes arising from Fe sublattice of TmFeO_3_ single crystal are characterized in a temperature range from 40 to 300 K, by using terahertz time-domain spectroscopy (THz-TDS). The magnetic anisotropy constants in *ac*-plane are estimated according to the temperature-dependent resonant frequencies of both FM and AFM modes. Here, we further observe the broad-band absorptions centered ~0.52, ~0.61, and ~1.15 THz below 110 K, which are reasonably assigned to a series of crystal-field transitions (R modes) of ground multiplets (^6^H_3_) of Tm^3+^ ions. Specially, our finding reveals that the spin reorientation transition at a temperature interval from 93 to 85 K is driven by magnetic anisotropy, however, which plays negligible role on the electronic transitions of Tm ions in the absence of applied magnetic fields.

Rare-earth orthoferrites (*R*FeO_3_ where *R* denotes Y and rare-earth ions (REI)) have the typical perovskite structure^1^,^2^ and possess two magnetic ions, rare earth *R*^3+^ and iron Fe^3+^ ions which crystallize in an orthorhombic lattice with 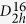-Pbnm space group^3^. In the past decades, ultrafast optomagnetic recording^4^,^5^,^6^,^7^, laser-induced thermal spin reorientation^8^,^9^, temperature and/or magnetic field induced spin switching and magnetization reversal^10^,^11^, spin modes resonant excitation and coherent control of magnetization dynamics by the magnetic field of terahertz pulses^12^,^13^,^14^,^15^,^16^,^17^,^18^ have been extensively studied in *R*FeO_3_. However, the study of the rare-earth electronic transition in the formation of the dynamic properties, which dominate the paramagnetic properties and the giant Faraday effect of these series of materials, should be further disclosed^19^,^20^,^21^,^22^.

Some *R*-ions, for instance Tm^3+^, Tb^3+^, Ho^3+^, are non-Kramers *R*-ions, which have an even number of the electrons and integer quantum number of the total angular momentum. The *R*-ions are split by crystal field into a series of singlets characterized by one of the two one-dimensional irreducible representations of the C_s_ group, A_1_ and A_2_. Optical spectra of the rare-earth ions in *R*FeO_3_ are characterized in primarily by the exchange interaction from iron ions as well as the crystal field. The isotropic exchange interaction is almost temperature-independence, which favors an antiferromagnetic configuration of the iron spins. While, the temperature-dependent magnetocrystalline anisotropy is the dominating mechanism for thermal-induced spin-reorientation transition (SRT), a rotation of the macroscopic magnetization by 90 degree, in *R*FeO_3_. Such a spin reorientation is thought to arise from temperature-induced repopulation of 4f-electrons in the rare-earth ions, which leads to a renormalization of the *R*-Fe interaction^1^. As a result, the temperature-induced SRT is expected to affect the optical properties of the *R*-ions in the crystal-field^20^. The thulium (Tm) is an even-electron ion with a series of isolated singlet states, the ground multiplets of Tm ions, ^6^H_3_, generated in the exchange field and crystal-field of TmFeO_3,_ has a strong absorption in terahertz frequency^21^,^22^, which allows the TmFeO_3_ single crystal as a good candidate for the investigation of SRT, crystal field transition (CFT) and Tm-Fe interaction.

Terahertz time-domain spectroscopy (THz-TDS) has been proved to be an effective tool to excite and probe the magnetic- and electronic-dipole transitions^23^,^24^,^25^. By using time-domain analysis, the amplitude and phase change of elementary excitations can be extracted. Such information is not available from conventional static spectroscopy^26^. To the best of our knowledge, the electronic excitations in *R*FeO_3_ with THz-TDS probe has been much less investigated^27^. Further explorations on the relevant subjects are indispensable to the growing of spintronics at frequencies reaching the terahertz regime.

In this study, we investigate the magnetic excitation of Fe ions as well as the electronic excitations of Tm ions in TmFeO_3_, in a broad temperature range from 40 to 300 K, by using THz-TDS. We discuss the magnetic thermodynamics of TmFeO_3_ single crystal using the temperature-dependent ferromagnetic and antiferromagnetic resonant frequencies of Fe ions sublattice. In addition, we observe a series of broad-band absorptions in the range from 0.2 to 1.5 THz, which are assigned to the crystal-field transitions of Tm ions systematically. SRT is determined by Tm-Fe interaction, while it plays a negligible role on the electronic transitions among the ground multiplets of Tm ions in TmFeO_3_ crystal, in absence of applied magnetic fields.

## Results and Discussion

**Figure 1:**
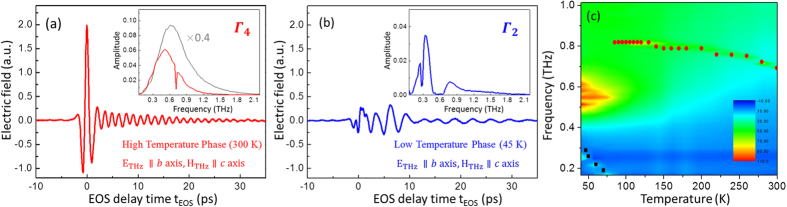
(**a**) The electric field of the THz wave passing through *a*-cut TmFeO_3_ single crystal at (**a**) high (300 K) and (**b**) low temperature phase (45 K) with E_THz_//b axis, H_THz_//c axis. Inset: the Fourier spectral amplitudes of the THz electric fields from the electro-optic sampling signals. The black curve in the inset of (**a**) is the THz spectrum without sample (dry nitrogen) (**c**) The amplitude mapping of absorption spectrum of the *a*-cut TmFeO_3_ crystal as a function temperature. The resonant frequencies of FM (square symbols) and AFM (circular symbols) modes are shown as functions of temperature.

Figure 1(a) shows the typical electric field of THz wave through the a-cut TmFeO_3_ single crystal at room temperature. The magnetic component of THz pulse is pointed along the *c*-axis of the crystal with Γ_4_ magnetic phase. At room temperature (high temperature phase), this configuration of *E*_*THz*_||*b, H*_*THz*_||*c*, allow us to excite a quasi-antiferromagnetic (AFM) mode. It corresponds to a narrow dip (Δω/ω_0_ ~ 0.01) observed at the resonance frequency of 0.71 THz in the Fourier amplitude spectrum, as shown in the inset of Fig. 1(a). At low temperature of 45 K, the TmFeO_3_ is transformed into Γ_2_ magnetic phase, i.e. the macroscopic magnetization is rotated from *c*-axis towards *a*-axis. In this case, the incident THz pulse was expected to excite the quasi-ferromagnetic (FM) mode, which has been seen as a narrow resonance dip at frequency of 0.28 THz, as shown the Fourier amplitude spectrum in the inset of Fig. 1(b). Apart from the FM mode at 0.28 THz, in particularly, it is noted that a broad absorption band (Δω/ω_0_~1) ranging from 0.4 to 0.65 THz is clearly observed. Figure 1(c) shows the amplitude mapping of absorption coefficient of *a*-cut TmFeO_3_ single crystal as a function of temperature, the broad-band absorption starts to be observable as the temperature below 110 K.

It is noted that the dispersion of refractive indices and absorption coefficients of the crystal can be calculated with the reference of THz transmittance in air. The calculation details are presented in [Supplementary Information 1], and it is seen that *Tm*FeO_3_ single crystal can be treated as a uniaxial crystal at room temperature with n_*a*_ ≈ n_*c*_ ~ 5.2 and n_*b*_ ∼ 4.6 in the 0.2~2.0 THz frequency regime.

**Figure 2:**
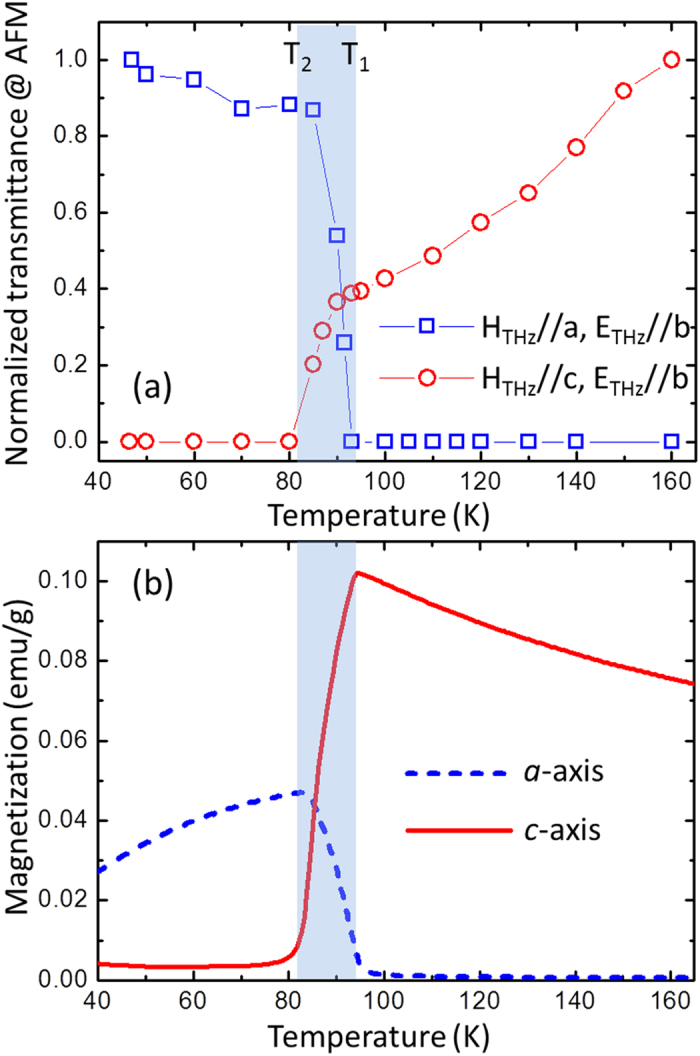
(**a**) Normalized amplitude of AFM mode as a function of temperature. The blue shade indicates spin reorientation transition temperature interval with higher and lower SRT temperatures of T_1_ = 93 K and T_2_ = 85 K, respectively. (**b**) Temperature dependence of macroscopic magnetization along *a*- (dashed) and *c*-axis (solid) of TmFeO_3_ single crystal.

We first look at the magnetic feature of the single crystal TmFeO_3_. The magnetic interaction between THz pulse and *R*FeO_3_ can be described with the Zeeman torque ***T*** = *γ**M*** × ***H***_*THz*_ with *γ* and ***H***_*THz*_ the gyromagnetic constant of the sample and impulsive magnetic field of the incident THz pulse, respectively^28^,^29^. According to our previous studies, the FM or AFM modes can be excited effectively with the ***H***_*THz*_ perpendicular or parallel to the macroscopic magnetization of the crystal, respectively^30^,^31^. In the temperature range of SRT, the macroscopic magnetization rotates continuously from *c*(*a*) towards *a*(*c*) axis, and then the amplitudes of both AFM and FM modes are expected to change with temperature, due to the cross production between vectors **M** and ***H***_*THz*_. Figure 2(a) shows the normalized amplitude of AFM mode as a function of temperature in a *c*-cut (blue squares) and *a*-cut (red dots) TmFeO_3_ single crystals. With the excitation geometry of ***H***_*THz*_ ⊥ *b* for the *c*-cut TmFeO_3_, AFM mode becomes activated below 93 K, and then increases slightly further below 85 K. While, for the *a*-cut crystal with the excitation geometry of ***H***_*THz*_ ⊥ *b*, AFM mode is activated in the high temperature region. The amplitude of AFM mode is seen to decrease gradually as the temperature decreases from 300 to 93 K, and then drops sharply and disappears completely below 85 K. Two critical temperatures can be obtained, T_1_ = 93 K and T_2_ = 85 K, corresponding to the SRT from Γ_4_ to Γ_24_ and Γ_24_ to Γ_2_, respectively. Our THz results are consistent with the temperature dependence of magnetization along *a*- (dashed) and *c*-axis (solid) of the crystal, respectively, as shown in Fig. 2(b).

**Figure 3:**
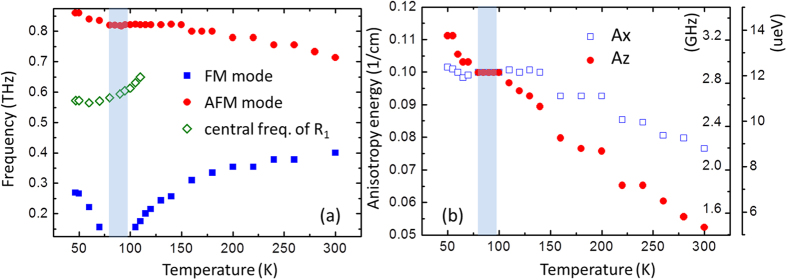
(**a**) Temperature dependence of resonance frequencies for FM mode (squares) and AFM mode (circles), as well as the center frequency of *R*_1_ mode (diamond). The shaded area shows the SRT temperature interval, within the magnetic mesophase Γ_24_. (**b**) Calculated magnetic anisotropy energies *A*_*x*_ and *A*_*z*_ in the *ac*-plane as a function of temperature.

Figure 3(a) shows the temperature dependence of magnetic resonance frequencies for both FM and AFM modes. It is clearly seen that the resonance frequency of AFM mode increase slightly with decreasing temperature. While, we find the softening of the FM mode as the temperature is approaching the spin reorientation range. SRT is driven by the temperature dependence of magnetic anisotropy change, which is mainly contributed by the *R*-Fe interaction^32^,^33^,^34^,^35^. The magnetic resonance frequency is related to the magnetic anisotropy constant in the *ac*-plane. *A*_*x*_ and *A*_*z*_ denote the magnetic constant along *x*(*a*) and *z*(*c*) axis, respectively. The relationship between magnetic resonance frequency and magnetic anisotropy can be expressed by^13^,^36^:


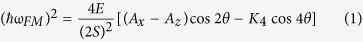



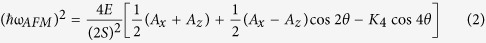


where *θ* is the angle between macroscopic magnetic moment and *z*-axis, the high-order magnetic anisotropy K_4_ is two-order smaller than that of *A*_*x*_ (*A*_*z*_). Figure 3(b) shows the temperature dependent anisotropy constants A_x_ and A_z_, calculated from the measured frequency data in Fig. 3(a) and Eqs (1) and (2). In one case, θ is almost zero at the high temperature phase Γ_4_ (above T_1_ = 93 K), the magnitude of *A*_*x*_ is larger than that of *A*_*z*_. As the case of crystal with low temperature phase Γ_2_ (below T_1_ = 85 K), θ is close to π/2. Thus, the magnitude of *A*_*z*_ is larger than that of *A*_*x*_. During the SRT temperature interval 85–93 K, *A*_*x*_ ≈ *A*_*z*_ = *A* (12.4 μeV), it leads to the completely soften of FM mode.

**Figure 4:**
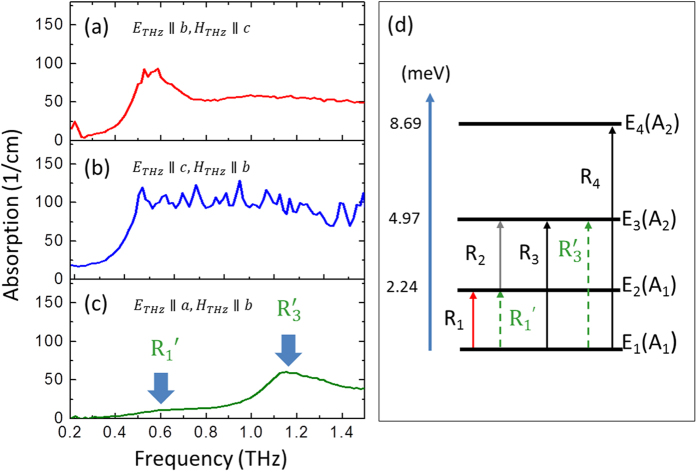
Absorption coefficient of TmFeO_3_ at 60 K with different excitation geometries (**a**) *H*_*THz*_||*c, E*_*THz*_||*b* in *a*-cut crystal, (**b**) *H*_*THz*_||*b, E*_*THz*_||*c* in *a*-cut crystal, and (**c**) *H*_*THz*_||*b, E*_*THz*_||*a* in *c*-cut crystal. (**d**) Energy diagram of ground multiplets of Tm ions.

Next, let us now discuss the physical origin of the broad absorption bands, which cannot be fully explained by the simple mechanism of THz spin modes excitation described above. Figure 4(a–c) shows the absorption spectra of TmFeO_3_ with different excitation configurations at 60 K. In retrieving the optical constants, the magnetic susceptibility μ ≈ 1 was assumed. This is justified by a much smaller contribution of the magnetic susceptibility to the complex refractive index compared to the dielectric one in orthoferrites^15^. With the excitation configuration of *H*_*THz*_||*c, E*_*THz*_||*b*, we find a nonsymmetrical absorption spectra, which has three contributions: (i) an isolated FM resonance feature according to the Γ_2_ phase, (ii) a broad absorption around 0.6 THz, and (iii) a dispersive feature covering the entire frequency window of our experiment. By rotating the sample 90 degree, the excitation configuration is changed to *H*_*THz*_||*b, E*_*THz*_||*c*. The THz absorption coefficient, as shown in Fig. 4(b), can be seen as a low pass filter characteristics at 60 K, which is significantly different from that in Fig. 4(a). We would like to mention that similar temperature dependence of the absorption is observed in a *b*-cut TmFeO_3_ crystal (see Supplementary Information 2). Furthermore, for the *c*-cut crystal with the excitation configuration of *H*_*THz*_||*b, E*_*THz*_||*a*, there have been two broad absorption modes at 60 K, as illustrated with arrows in Fig. 4(c). Notably, the absorption magnitude around 0.53 and 1.15 THz are much weaker, compared with that of Fig. 4(a). All detailed temperature dependence of THz transmission spectra is presented in Supplementary Information. Taken together, our observations reveal that the absorption are strongly dependent on the electric field orientation of the incident THz pulse and the crystal orientation. Therefore, they are assigned to the electronic transitions inside the ground multiplets of the Tm ions at low temperature regime.

Figure 4(d) shows the energy diagram of the ^6^H_3_ ground multiplets of Tm^3+^ ions, which is split into series of singlets in the low symmetry crystal field of the TmFeO_3_ single crystal. A_1_ and A_2_ denote for the irreducible representations of the symmetry point group *C*_*S*_ for these singlets. Due to non-centrosymmetric positions occupied by Tm ions in the orthoferrite structure, these transitions between states within the ground multiplets of Tm ions are both electrodipole and magnetodipole allowed^21^,^22^,^37^. The *R*_1_ mode is active with excitation configuration of *H*_*THz*_||*c* and *E*_*THz*_||*b*, which is corresponding to the transition between *E*_1_(*A*_1_) and *E*_2_(*A*_1_) singlets, as illustrated as red arrow in Fig. 4(d). *R*_2_ mode can be activated with *H*_*THz*_||*b, E*_*THz*_||*c*, corresponding to the electronic transition from *E*_2_(*A*_1_) to *E*_3_(*A*_2_). However, our observed absorption band (see Fig. 4(b)) is very broad, the low pass filter characteristic makes it hard to determine the absorption peak of *R*_*2*_ mode accurately. This is due to the following reasons: firstly, under the excitation configuration *H*_*THz*_||*b, E*_*THz*_||*c*, both *R*_*2*_ mode (E_2_ → E_3_) and *R*_*3*_ mode (E_1_ → E_3_), as well as *R*_*4*_ mode (E_1_ → E_4_) are activated. Remarkably, these *R* modes have broad bandwidth and large absorption cross section, which is proved by approaching saturable absorption when the temperature is as low as 80 K. (see Supplementary Information 3). Secondarily, the THz transmittance of *R*FeO_3_ is relatively lower at higher frequency due to the dielectric anisotropy of orthoferrite caused by phonon (see Supplementary Information 1). Two weak absorption modes, as shown in Fig. 4(c) at low and high frequencies are assigned as 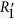 and 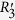 modes, corresponding to the electronic transitions *E*_1_(*A*_1_) → *E*_2_(*A*_1_) and *E*_1_(*A*_1_) → *E*_3_(*A*_2_), respectively. We note that the transition probability of 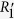 mode is much weaker than that of *R*_1_ mode. Moreover, 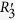 mode undergoes a frequency shift with decreasing temperature, and the absorption peak shifts from 1.15 THz at 80 K to 1.4 THz at 45 K (see Supplementary Information 3).

**Figure 5:**
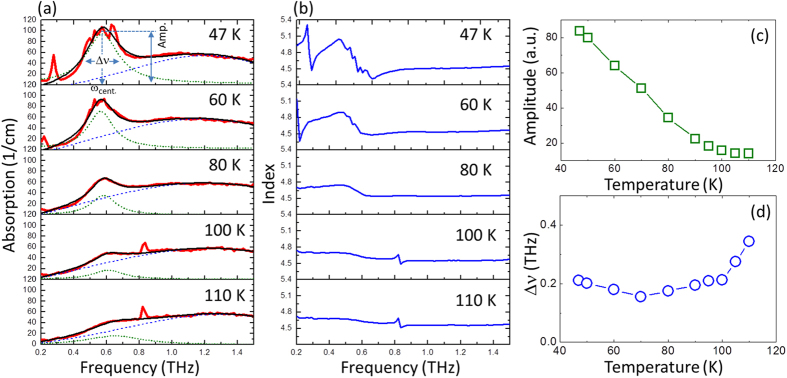
(**a**) Absorption coefficient and (**b**) refractive index of *a*-cut TmFeO_3_ single crystal under various temperatures. Solid lines in (**a**): Bi-Lorentz spectra line fits, see text for details. Temperature dependence of (**c**) amplitude and (**d**) bandwidth Δν of R_1_ mode.

For now, let us concentrate on the absorption coefficients and refractive indices of our *a*-cut crystal at various temperatures, as shown in Fig. 5(a,b), respectively. The magnitude of absorption coefficient and dispersion of refractive index become more pronounced with decreasing temperature. To gain insight into the *R*_*1*_ mode, the bi-Lorentz spectra fitting routine is employed as a global fit (solid lines) to the absorption spectra, in absence of the isolated spin resonance, with two component spectra: (1) a common temperature-independent spectrum from background absorption of orthferrite at high frequency (blue dashed line). (2) a spectrum of *R*_*1*_ itself (green dots). Here, Δν is defined as the frequency difference at half maximum of the amplitude, as marked in Fig. 5(a). The temperature dependent amplitude and Δν are shown in Fig. 5(c,d). The amplitude increases with decreasing temperature, which can be well explained with two energy levels system. According to Boltzmann distribution, 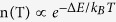 with ΔE = *E*_2_ − *E*_1_, the energy difference between the two levels, and the Boltzmann constant k_B_. At lower temperature, the carrier densities for the energy level *E*_2_ is much smaller than that for E_1_ at thermal equilibrium. Therefore, more THz photons can be absorbed by the Tm ions at lower temperature. On the other hand, Δν decreases from 0.34 to 0.20 THz with decreasing temperature from 110 to 70 K. As the temperature decreases further below 70 K, Δν can be regarded as nearly temperature independent, at around 0.2 THz. Combining the observed bandwidth narrowing with the Lorentz-type line shape fitting, the *R*_*1*_ mode was reasonable to demonstrate a homogeneously broadening in nature. The broadening mainly comes from the interaction between the Tm ions and phonons in the crystal. We can also comment that the impurities scattering has negligible influence on the *R*_1_ mode of Tm ions. *R*_1_ mode arises from the resonant transition between *E*_1_ and *E*_2_ singlet states generated by the crystal field and exchange field of TmFeO_3_. In terms of the isotropic exchange interaction is almost temperature-independent, hence the origin of *R*_1_ mode is attributed to the temperature dependent crystal field.

The temperature dependence of central frequency of Tm-ions absorption is shown in Fig. 3(a). It can be clearly seen that the central frequency ν_cent_ decreases slightly over the temperature range from 110 to 40 K. In the temperature regime of SRT, there are no dramatic changes of the amplitude, Δν and ν_cent_. In particular, we do not observe any anticrossing behavior of *R*_1_ and AFM mode in the present experimental condition. Our results can be readily to suggest that magnetocrystalline anisotropy, the driving force of SRT, has a negligible contribution to the crystal-field transition in TmFeO_3_. Thus, in the absence of the applied magnetic field and at temperature well below 110 K, the SRT and crystal-field transition of Tm ions take place independently. Moreover, we note an interesting splitting of the absorption band into multiple branches, occurred at 47 K, as shown in Fig. 5(a). Further work is ongoing in our group to reveal the hyperfine structure of the crystal field transitions with increasing magnetic field.

## Conclusions

In summary, we have investigated the spin reorientation transition and crystal-field transition in the TmFeO_3_ single crystal using THz time domain spectroscopy, over a wide temperature range from 40 K to 300 K. The temperature dependence of the magnetic anisotropy constants in *ac*-plane have been estimated from the frequencies of FM and AFM modes. In addition, the electronic transitions among the ground multiplets of Tm ions has been observed at the THz range. Current results suggest that the magnetic anisotropy has a negligible influence on the *R* modes characteristics at low temperature range. Further THz absorption coefficient spectra at varying magnetic fields are warranted for the studies of hyperfine structure of the TmFeO_3_ single crystal.

## Methods

### Sample preparation

The TmFeO_3_ single crystal in our study were grown by the floating zone method. The samples with *a*-, *b-* and *c-* cut plane parallel plates having thickness of 1.16, 0.89 and 1.31 mm respectively, were polished on both sides, The directions of the crystal axes were determined by the x-ray Laue analysis. For the growth of TmFeO_3_ single crystal, we started with a stoichiometric mixture of Tm_2_O_3_ (99.9%) and Fe_2_O_3_ (99.99%) powders that was calcined at a temperature of 1200 °C for 12 h in air. The milled presintered material was isostatically pressed into a cylindrical rod of 120 mm length and 8 mm diameter under150 MPa, and the rod was sintered at 1300 °C for 12 h. Then we reground, pressed and sintered the obtained rod again under the same conditions in order to obtain high-quality polycrystallite. A TmFeO_3_ single crystal was successfully grown in a four-mirror optical floating-zone furnace (FZ-T-10000-H-VI-P-SH, Crystal Systems Corp.) using four 1.5 kW halogen lamps as the infrared radiation source with flowing air. The temperature of the molten zone focused by mirrors was precisely controlled by adjusting the power of the lamps. During the growth process, the molten zone moved upwards at rates of 3 mm/h, with the seed rod (lower shaft) and the feed rod (upper shaft) counter rotating at 30 rpm in air flow of 5 L/h. The orientations of the as-grown crystal prepared and crystalline qualities were analyzed by X-ray Laue analysis (see Supplementary Information 4).

### THz-TDS experimental setup

THz-TDS measurements in transmission configuration were conducted on the TmFeO_3_ single crystal in the frequency range between 0.1 and 2.0 THz over a temperature range from 40 to 300 K. Briefly, the output of a mode-locked Ti: Sapphire laser, with pulse duration of 100 fs, centered wavelength of 800 nm, and repetition rate of 80 MHz (Mai Tai HP-1020, Spectra-Physics), was used to generate and detect the THz transient. The emitter and detector of the THz wave were photoconductive antennas fabricated on low-temperature-grown GaAs substrate. The polarization of the THz radiation was horizontal, which was perpendicular to the photoconductive antennas. No external static magnetic field was applied on the sample during the measurement. The sample was installed in a cold finger cryostat with two THz transparent windows, of which the temperature is tunable in a range from 40 to 300 K with best resolution of 1 K.

## Additional Information

**How to cite this article**: Zhang, K. *et al*. Resolving the spin reorientation and crystal-field transitions in *Tm*FeO_3_ with terahertz transient. *Sci. Rep.* **6**, 23648; doi: 10.1038/srep23648 (2016).

## Supplementary Material

Supplementary Information
