# Supplementary material for: Resolving the spin reorientation and crystal-field transitions in TmFeO3 with terahertz transient
Source: Sci Rep. 2016 Mar 24;6:23648. doi: 10.1038/srep23648 (PMC4806309; doi:10.1038/srep23648)
Supplement: Supplementary Information [file srep23648-s1.pdf]

Supplementary Information

Resolving the spin reorientation and crystal-field transitions in $TmFeO_3$ with terahertz transient

Kailin Zhang[1], Kai Xu[1], Xiumei Liu[1], Zeyu Zhang[1], Zuanming Jin[1*], Xian Lin[1], Bo Li[2], Shixun Cao[1], and Guohong Ma[1*],

[1] *Department of Physics, Shanghai University, Shanghai 200444, China.*

[2] *Key Laboratory of Polar Materials and Devices, East China Normal University, Shanghai 200241, China*

* Corresponding author: Z. J (physics_jzm@shu.edu.cn) and G. M (ghma@staff.shu.edu.cn)

1. The extraction of optical parameters

At room temperature, the absorption due to Tm ions is negligible, and the magnetic resonance due to iron ions play dominating role in the 0.2~2.0 THz spectra range. The transmitted THz pulses through the sample and the reference (dry nitrogen) were recorded in the time domain. The complex transmittance $\tilde{T}(\omega,\varphi)$ can be expressed as:

$$\tilde{T}(\omega,\varphi)=\frac{E_{sample}}{E_{reference}}=Ae^{i\varphi}$$

$$=\frac{4n(\omega)}{(n(\omega)+1)^2}\exp(-\kappa(\omega)\omega d/c)\times\exp(i(n(\omega))-1)\omega d/c) \qquad (S1)$$

The E_{sample} and $E_{reference}$ are the transmitted THz electric fields through sample and air, respectively, and A and φ denote the amplitude ratio and phase shift extracted from the Fourier transform from the time-domain spectra. The complex refractive index of the sample is $\tilde{n}=n(\omega)-ik(\omega)$ with refractive index $n(\omega)$ and extinction coefficient $k(\omega)$. The absorption coefficient is $\alpha(\omega)=2\omega k(\omega)/c$ with c the speed of light in vacuum. After considering the sample thickness d, the refractive index and the absorption coefficient can be obtained from the time domain spectra, which read:

$$n(\omega)=1+\frac{\varphi c}{\omega d} \qquad (S2)$$

$$\alpha(\omega)=\frac{2}{d}In\left[\frac{A(\omega)(n(\omega)+1)^2}{4n(w)}\right] \qquad (S3)$$

We calculated the refractive indices and absorption coefficients at room temperature based on the above equations. The calculated refractive indices and absorption coefficients along *a*-, *b*-, and *c*-axis of the crystal are presented in Fig. S1. It is interesting to note from Fig. S1 (a) that refractive indices along *a*- and *c*-axis, i.e. n_a and n_c, show similar magnitude with $n_a \approx n_c \sim 5.2$, but the refractive index along *b*-axis, $n_b \sim 4.6$, is much smaller than n_a and n_c, refractive index difference along *a*-(*c*-) and *b*-axis of the crystal, $\Delta n = n_a(n_c) - n_b$, is as large as ~0.6. Therefore, the $TmFeO_3$ can be treated as a uniaxial crystal in the 0.2~2.0 THz frequency regime. The larger refractive indices n_a and n_c may come from the strong phonon absorption of the $TmFeO_3$ crystal along *a*- and *c*-axis. Figure S1 (b) shows the absorption coefficients along *a*-, *b*- and *c*-axis in the frequency range 0.2~1.5 THz, it is seen that the absorption

coefficient along *a*- and *c*-axis is much stronger than that along *b*-axis, which is consistent with the refractive index data of the crystal. The larger coefficient along *a*- and *c*-axis than that along *b*-axis indicates that the phonon modes along *a*- and *c*- are different from that along *b*-axis. Our far infrared Fourier reflection spectra reveal that the lowest phonon mode in $TmFeO_3$ is located around ~5.7 THz for all-cut crystals, but the magnitude of reflection dip around 5.7 THz for the *b*-cut sample is ~29%, which is larger than that for *a*-cut (~22%) and *c*-cut (~17%) samples. Due to light source in our FTIR is nonpolarized, the reflection dips for *a*-, *b*- and *c*-cut samples only give the average absorbance in (*bc*)-, (*ac*)- and (*ab*)-plane, respectively. For quantitative analysis of the phonon mode along the three axes, polarized far IR reflection have to be measured. From these data, we can conclude that the relatively smaller refractive n_b is due to the weak phonon absorption along *b*-axis of the crystal.

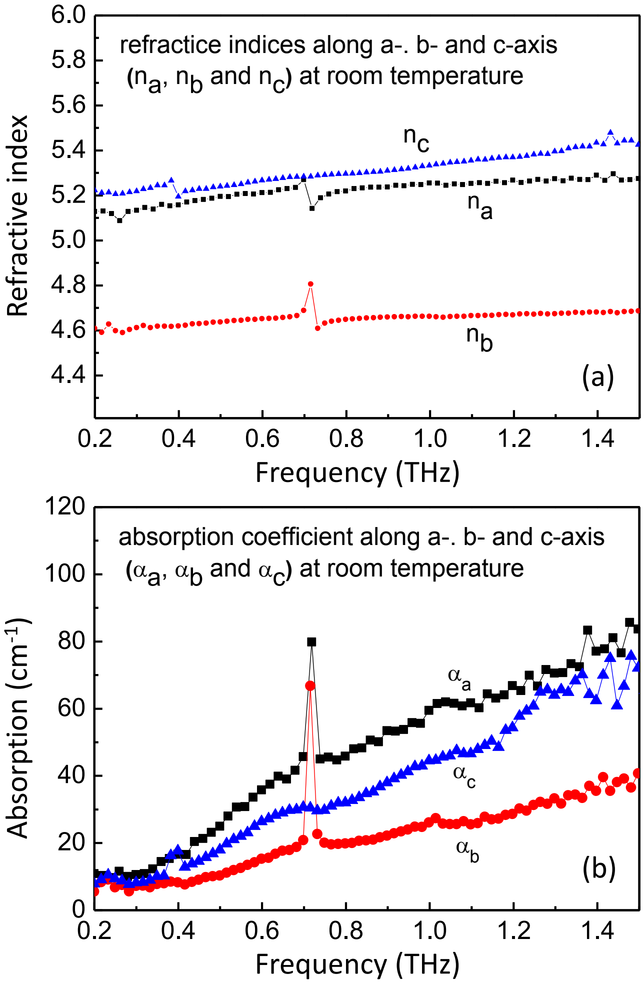

Figure S1 Dispersion of refractive indices (a) and absorption coefficients (b) along *a*-, *b*- and *c*-axis of the $TmFeO_3$ crystals at room temperature.

2. Temperature dependence of terahertz Fourier amplitude spectrum under various experiment geometries

In order to obtain THz absorption information about iron and thulium ions as a function of temperature, the temperature dependence of THz Fourier amplitude spectrum as well as the amplitude mapping of the THz transmittance are displayed in the Fig. S2 (E ∥ c,H ∥ b), Fig. S3 (E ∥ a, H ∥ c and E ∥ c, H ∥ a) and Fig. S4 (E ∥ a, H ∥ b and E ∥ b, H ∥ a). The FM and AFM modes are highlighted with solid red circles and solid blue squares, respectively. Apart from FM and AFM absorptions, series of broad band absorptions are observed, which are assigned to R_1-mode (Fig. S3 (a), S3 (b)) and R_2-mode (Fig. S2 (a), S2 (b) and Fig. S3 (c), S3 (d)) of Tm ions. With excitation of E ∥ a, H ∥ b and E ∥ b, H ∥ a) shown in Fig. S4, the absorptions (R_1'- and R_3'-modes) are much weaker than those of R_1- and R_2-modes.

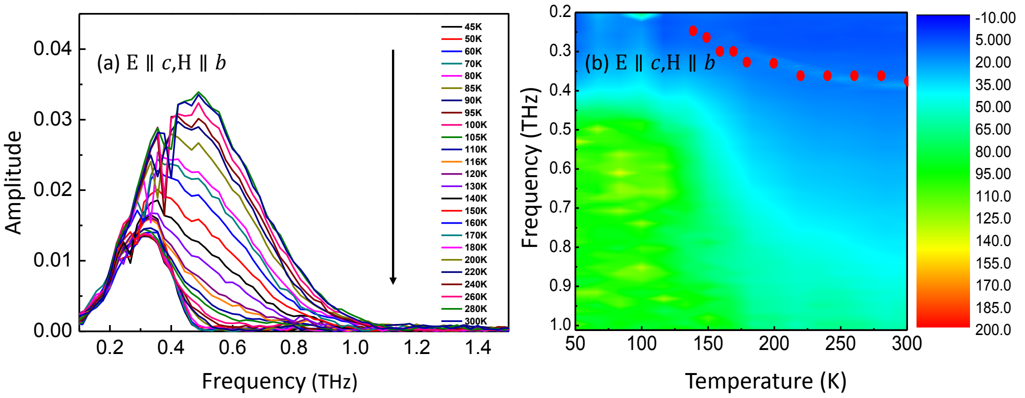

Figure S2 THz Fourier amplitude spectra for a-cut $TmFeO_3$ in the temperature range from 40 K to 300 K. The spectra with the excitation configuration of E ∥ c,H ∥ b (a), and the 2D plots (frequency vs. temperature) of (a) are presented in (b). The arrows marked in (a) is denoted for the decrease of temperature.

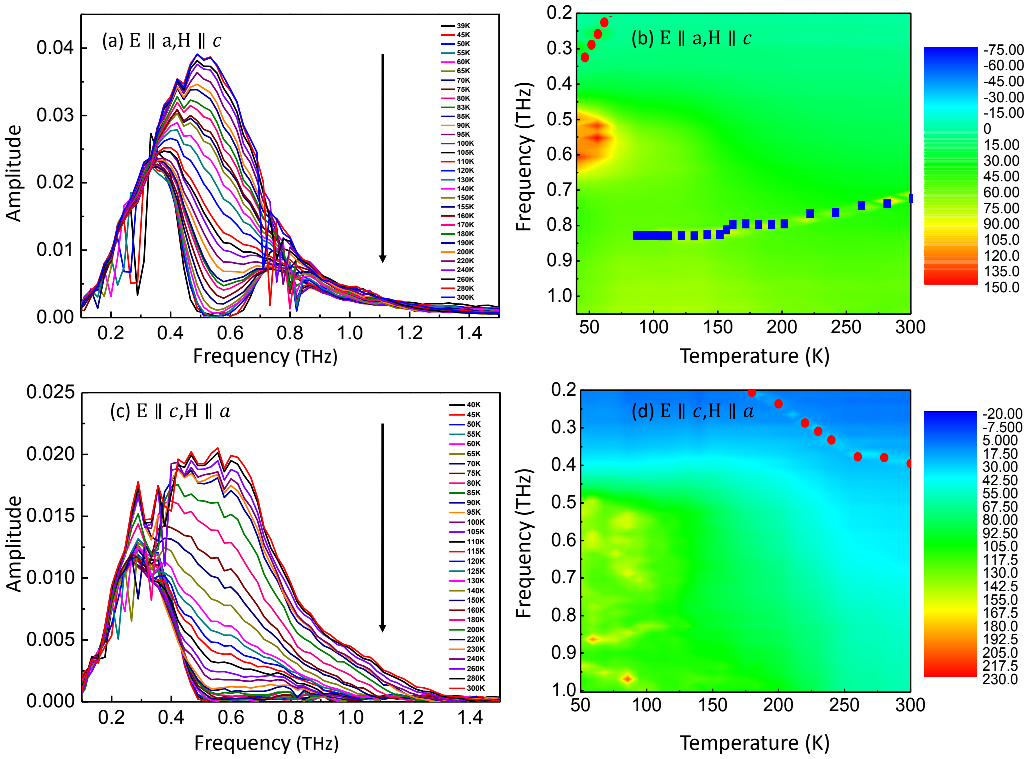

Figure S3 THz Fourier amplitude spectra for *b*-cut $TmFeO_3$ in the temperature range from 40 K to 300 K. The spectra are obtained with the excitation configuration of E ∥ a,H ∥ c (a) and E ∥ c,H ∥ a (c), and the 2D plots (frequency vs. temperature) of (a) and (c) are presented in (b) and (d), respectively. The arrows marked in (a) and (c) are denoted for the decrease of temperature.

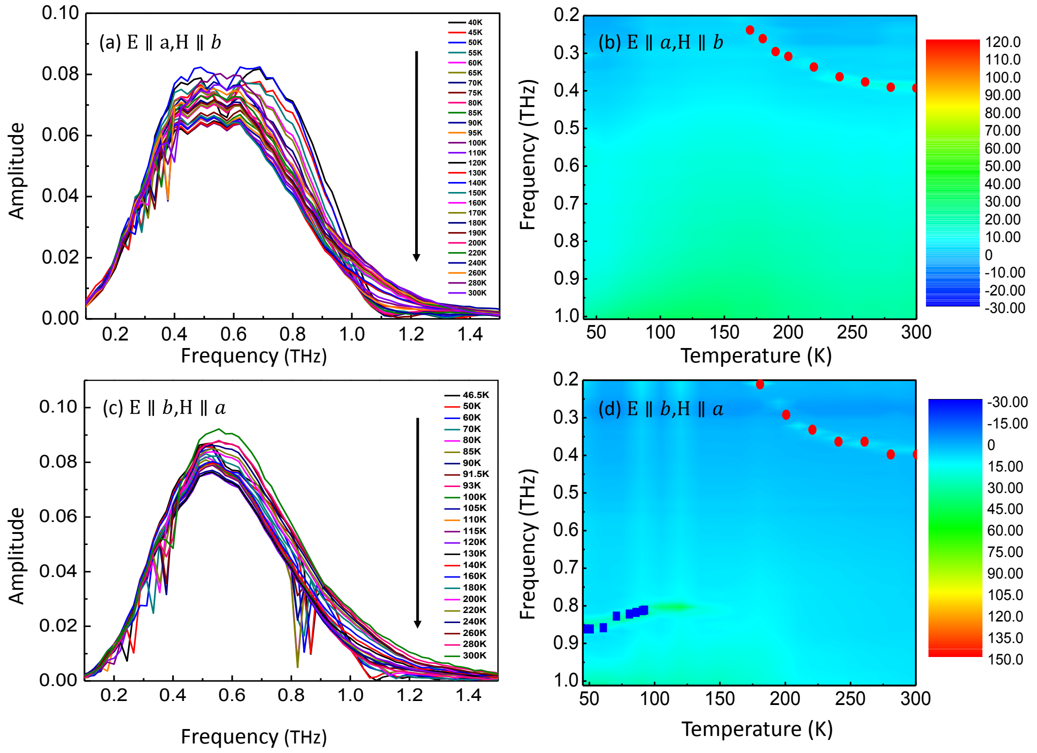

Figure S4 THz Fourier amplitude spectra for *c*-cut $TmFeO_3$ in the temperature range from 40 K to 300 K. The spectra are obtained with the excitation configuration of $E \parallel a, H \parallel b$ (a) and $E \parallel b, H \parallel a$ (c), and the 2D plots (frequency vs. temperature) of (a) and (c) are presented in (b) and (d), respectively. The arrows marked in (a) and (c) are denoted for the decrease of temperature.

3. The broad band absorption coefficients with different excitation in the $TmFeO_3$ single crystals

In addition to the R_1-mode absorption, under the excitation configuration of $E_{THz} \parallel c$, $H_{THz} \parallel b$ in an *a*-cut crystal, the R_2-mode is active, and Fig. S5 presents the calculated absorption coefficients at various temperatures for the R_2-mode. For the excitation configuration of $E_{THz} \parallel a$, $H_{THz} \parallel b$ in a *c*-cut crystal, the R_1'- and R_3' modes can be excited, and Fig. S6 shows the calculated absorption coefficients of these two modes at temperatures of 45, 60 and 80 K, respectively.

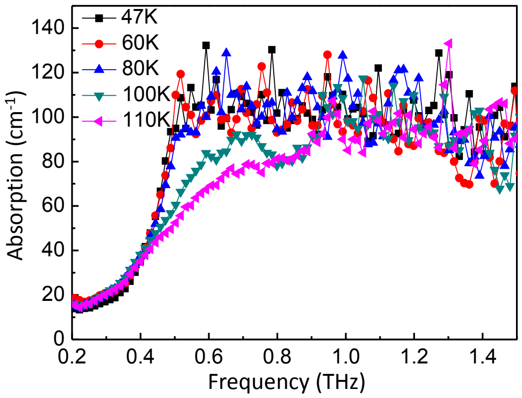

Figure S5 Absorption coefficient for R_2-mode at various temperature with excitation of $E_{THz} \parallel c$, $H_{THz} \parallel b$ in an *a*-cut $TmFeO_3$ crystal.

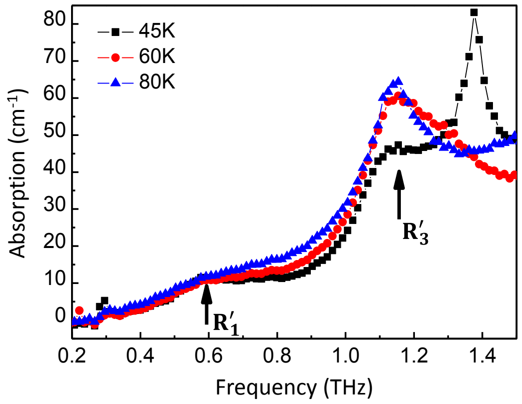

Figure S6 Absorption coefficient for R_1'-mode and R_3'-mode at various temperature with excitation of $E_{THz} \parallel a$, $H_{THz} \parallel b$ in a *c*-cut $TmFeO_3$ crystal, two weak modes assigned as R_1'-mode and R_3'-mode are indicated with arrows.

4. The Laure diffraction pattern of $TmFeO_3$ single crystal.

The $TmFeO_3$ single crystal was grown in a four-mirror optical floating-zone furnace using four 1.5 kW halogen lamps as the infrared radiation source with flowing air. The samples with *a*-, *b*- and *c*-cut are orientated by using X-ray Laue photography.

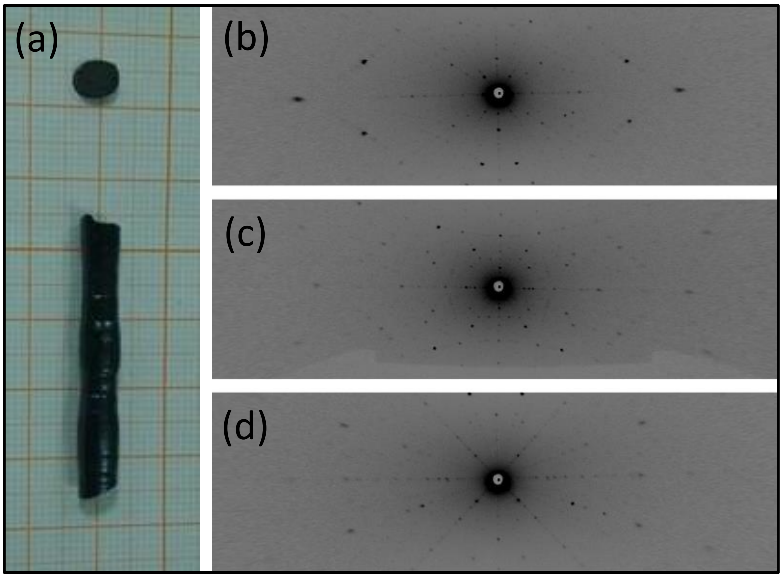

Figure S7. (a) The photograph of the as-grown $TmFeO_3$ single crystal rod, and (b) to (d) display the X-ray Laue diffraction patterns for the *a*-, *b*- and *c*-cuts of $TmFeO_3$ crystals used in this study, respectively. The Laue patterns were taken at room temperature.
